# Type 2 Diabetes and all-cause mortality among Spanish women with breast cancer

**DOI:** 10.1007/s10552-021-01526-x

**Published:** 2021-12-01

**Authors:** L. Fernández-Arce, N. Robles-Rodríguez, A. Fernández-Feito, A. Llaneza-Folgueras, A. I. Encinas-Muñiz, A. Lana

**Affiliations:** 1 Community Health Center of La Ería, Health Care Service of Asturias, C/Alejandro Casona s/n, 33013 Oviedo, Spain; 2 Department of Medicine, School of Medicine and Health Sciences, University of Oviedo, Avda Julián Clavería s/n., 33006 Oviedo, Spain; 3 Institute of Sanitary Research of Asturias (ISPA), Avda de Roma s/n, 33011 Oviedo, Spain; 4 Breast Pathology Unit, Health Care Service of Asturias, Central University Hospital of Asturias, Avda de Roma s/n, 33011 Oviedo, Spain; 5 Anatomical Pathology Service, Health Care Service of Asturias, Central University Hospital of Asturias, Avda de Roma s/n, 33011 Oviedo, Spain

**Keywords:** Breast neoplasms, Diabetes mellitus, Type 2, Survival analysis, Follow-up studies, Female

## Abstract

**Purpose:**

To explore the effect of type 2 diabetes mellitus (T2DM) on the risk of death among women with breast cancer (BC).

**Methods:**

A survival analysis was conducted among a cohort of women diagnosed with BC between 2006 and 2012 in Spain (*n* = 4,493). Biopsy or surgery confirmed BC cases were identified through the state population-based cancer registry with information on patients’ characteristics and vital status. Physician-diagnosed T2DM was confirmed based on primary health care clinical history. Cox regression analyses were used to estimate adjusted hazard ratios (aHR) and 95% confidence intervals (CI) for all-cause death. Analyses were adjusted for age, hospital size, several clinical characteristics (including BC stage and histology, among others) and treatment modalities.

**Results:**

Among the 4,493 BC women, 388 (8.6%) had coexisting T2DM. Overall, 1,299 (28.9%) BC women died during the completion of the follow-up and 785 (17.5%) did so during the first five years after BC diagnosis, resulting in a five-year survival rate of 82.5%. The death rate was higher in women with T2DM (43.8% died during whole period and 26.0% during the first five years) when compared with women without T2DM (27.5% and 16.7%, respectively). Accordingly, all-cause mortality was higher in women with T2DM (aHR: 1.22; 95% CI 1.03–1.44), especially if T2DM was diagnosed before BC (aHR:1.24; 95% CI 1.03–1.50) and in women with BC diagnosed before 50 years (aHR: 2.38; 95% CI 1.04–5.48).

**Conclusions:**

T2DM was associated with higher all-cause mortality among Spanish women with BC, particularly when the T2DM diagnosis was prior to the BC.

**Supplementary Information:**

The online version contains supplementary material available at 10.1007/s10552-021-01526-x.

## Introduction

Breast cancer (BC) is the most commonly diagnosed malignancy worldwide, even considering both sexes combined [1, 2]. After years of rising mortality rates, in the last decades BC survival has improved in most European countries, mainly due to advances in therapy and to the efficacy of screening programs for early diagnosis [3, 4]. In Spain, the five-year relative survival in 2000–07 was 82.8%, which meant an increase of 2.4 percent concerning the last decade of the twentieth century [5]. Likewise, Clèries et al. [6] found a 19 percent reduction in the 10-year risk of death of women diagnosed with BC in 1995–2004 compared to women diagnosed in 1985–1994. Moreover, according to current estimates on mortality by the International Agency for Research on Cancer, Spain is the European country with the lowest age-standardized BC death rates [2]. Nevertheless, there is still room for improvement in BC survival.

There is a growing body of evidence linking type 2 diabetes mellitus (T2DM) and BC. Both are diseases associated with aging that share a wide variety of risk factors, including socioeconomic conditions, lifestyle behaviors, and body fat [7, 8]. Moreover, T2DM and BC have been hypothesized to be causally associated through the tumorigenic effect of hyperinsulinemia, insulin-like growth factors, and other hormones [9, 10]. Several meta-analyzes have found a pooled 15–20% excess of risk of BC among women with preexisting T2DM [11–13], even adjusting for overweight/obesity, which is the main shared risk factor. Specifically, a preexisting T2DM has been found to increase the risk of BC with poor prognosis [14]. Accordingly, there is also strong evidence from studies involving different countries and races about the increased risk of death among women with BC and preexisting T2DM, when compared with women with BC, but who are T2DM-free [15–18]. Nevertheless, the impact of T2DM on mortality among women with BC has not been adequately studied in Spain. Indeed, the only study identified was published in 2019 and, although some findings were reported about the association between T2DM and BC, the study was not specifically focused on these factors. In a cohort of 7.338 women with BC diagnosed from 1985–2004 in Girona (Spain), Ameijide et al. [19] found a 1.43 standardized mortality ratio among women with T2DM, after a 10-year follow-up.

Therefore, to examine the consistency of these results and determine the effect of the timing of the T2DM diagnosis, a study was conducted to explore the effect of T2DM (preexisting or subsequent) on the risk of death among women with BC.

## Subjects and methods

### Study design and participants

A survival analysis was conducted among a cohort of women diagnosed with BC between 2006 and 2012 in Asturias (Spain) and retrospectively followed-up until the end of 2019. Data were obtained from the state population-based cancer registry, which is one of the oldest established cancer registries in Spain, and which collaborates with the International Agency for Research on Cancer by cancer data provision.

An anonymous database was created in STATA v.15 (StataCorp, College Station, Texas), with units working with biopsy or surgery confirmed BC cases (*n* = 4,704 cases, after excluding 56 cases of BC among males). Subsequently, to avoid any overestimation due to multiple BC on survival, a single case of BC was selected per woman, by excluding all but one case of BC diagnosed in the same woman. When a woman had two or more diagnoses of BC, the first diagnosed case of BC was selected if both cases were metachronous (i.e., time between diagnoses > 6 months), or, the case with the worse prognosis was selected (i.e. more advanced stage and/or greater treatment intensity) if they were synchronous.

The study protocol was approved by the Research Ethics Committee of Asturias (Spain). The study was performed in accordance with the ethical standards of the 1964 Declaration of Helsinki and its subsequent amendments.

### T2DM and mortality ascertainment

First, we merged the database from the cancer registry with the computerized clinical history of primary healthcare (OMI-AP software) to identify BC women with an additional physician-diagnosed T2DM. Subsequently, we performed a computerized search of the National Death Index to evaluate all-cause mortality. This database contains updated information on the vital status of all residents in Spain. Information regarding death date after 2006 was available for 99.9% of the cohort. Censoring was set at the date of death or at the end of follow-up (31 December 2019), whichever occurred first.

### Covariates

Covariates were selected by combining two criteria. On the one hand, these should be variables linked to either T2DM or mortality according to scientific literature. On the other hand, they should be variables for which information is available on the administrative databases used in this study (i.e., cancer registry, OMI-AP software and National Death Index) and with the possibility of automatic data extraction. Given that some important predictors of death, such as body weight and comorbid conditions, were only available in plain text on health records (and not stated in all records), their inclusion would have implied both an exhaustive manual review and a reduction of the study population. Therefore, this information was not considered in the study.

The study variables included age at BC and T2DM diagnosis, hospital size, measured by number of beds (< 500, ≥ 500), BC suspected via a population screening program (yes/no), another non-BC cancer (yes/no), BC staging and histology were coded according to the American Joint Committee on Cancer and the International Classification of Diseases for Oncology, 3rd edition (ICD-O-3) [20, 21], BC location (breast quadrants, central part of the breast, nipple, axillary extensions, contiguous sites, unspecified sites) and types of treatments, including surgery, chemotherapy, radiotherapy, hormone therapy, immunotherapy, targeted therapy, among others.

### Data analysis

Of the 4,704 BC cases included in the study, 37 were excluded because they were duplicates, suggesting coding errors, and 164 were excluded because they were multiple BC cases (27 metachronous and 137 synchronous). Of the remaining 4,503 women with BC, 10 were excluded due to missing information on the national identity document, and consequently, a lack of information on survival status. Finally, the analyses were conducted in 4,493 women with BC.

Cox proportional-hazards models were used to assess the association between T2DM and mortality during the first five years of follow-up and mortality throughout the entire period among women with BC (mean years of follow-up was 8.66). Additionally, to study the effect of the time of T2DM diagnosis on survival, we performed two analyses with T2DM, classified into categories. The first one used two categories (diagnosis of T2DM before or after BC) whereas the second used three categories (diagnosis of T2DM > 5 years before BC, 0–5 years before BC and after BC). Women without T2DM were always used as the reference. In all cases, we built two Cox models, the first one was crude, whereas the second one was adjusted. Previously, a bivariate analysis was used to identify the variables related to both T2DM and death, to consider those with a p-value < 0.2 as potential confounders in the adjusted model. The variables included in the adjusted model were age at BC diagnosis (< 50, 50–59, 60–69, ≥ 70 years), hospital size (< 500, ≥ 500 beds), BC suspected via screening (yes/no), other non-BC cancer (yes/no), BC stage (0-I, II, III-IV, not applicable, unknown), BC histology (non-invasive ductal carcinoma, invasive ductal carcinoma, non-invasive lobular carcinoma, invasive lobular carcinoma, other ductal and lobular, other types), BC localization (any breast quadrant, central part/nipple, axillary/contiguous sites, unspecified site) and types of BC treatments, including surgery, chemotherapy, radiotherapy, hormone therapy, immunotherapy, targeted therapy and others. Given that there were many possible combinations, BC treatment modalities were used as dichotomous variables for data analysis. Finally, we used the Kaplan–Meier method to estimate the unadjusted survival function and the log-rank test to compare survival curves. Statistical significance was set at *p* < 0.05.

## Results

Among the cohort of women with BC (*n* = 4,493), 388 (8.6%) had a physician-diagnosed T2DM. In three out of four women (75.3%) T2DM was diagnosed before BC. Statistically significant differences between women with and without T2DM were only found for age of BC and treatment types. Compared to women without T2DM, those with T2DM were older (*p* < 0.001) and had lower intensity treatment, as they received more hormone therapy (*p* = 0.018), although less surgery (*p* = 0.001), chemotherapy (*p* < 0.001), radiotherapy (*p* = 0.023) and targeted therapy (*p* = 0.003) (Table 1).

**Table 1:** Clinical characteristics of BC patients according to T2DM diagnosis

	No T2DM	T2DM	*p*-value^a^
Participants, *n*	4,105	388	
Age at BC diagnosis, y	60.1 (14.4)	69.6 (11.2)	< 0.001
Age at T2DM diagnosis, y	NA	65.5 (11.2)	
Time of T2DM diagnosis, %			
> 5 years before BC	NA	34.3	
0–5 years before BC	NA	41.0	
After BC	NA	24.7	
Hospital, %			0.807
≥ 500 beds	16.8	16.3	
< 500 beds	83.2	83.7	
BC suspected via screening, %	16.3	14.2	0.413
Multiple cancer, %	13.8	15.0	0.483
Stage, %			0.085
Stages 0-I	53.1	57.0	
Stage II	32.3	29.9	
Stages III-IV	5.26	6.96	
Not applicable	7.77	4.64	
Unknown	1.66	1.55	
Histology, %			0.309
Non-invasive ductal carcinoma	6.97	4.12	
Invasive ductal carcinoma	76.1	80.4	
Non-invasive lobular carcinoma	0.44	0.26	
Invasive lobular carcinoma	7.94	7.47	
Other ductal and lobular	4.19	3.61	
Other types	4.41	4.12	
Localization, %			0.320
Breast quadrants	53.2	52.8	
Central part / Nipple	7.09	9.02	
Axillary / Contiguous sites	30.9	30.7	
Unspecified site	8.82	7.47	
Treatment, %			
Surgery	89.4	83.5	0.001
Chemotherapy	36.1	24.4	< 0.001
Radiotherapy	58.1	51.8	0.023
Hormone therapy	47.4	53.9	0.018
Immunotherapy	0.15	0.26	0.597
Targeted therapy	5.46	1.80	0.003
Other therapy	0.37	0.52	0.773

*T2DM* type 2 diabetes mellitus, *BC* breast cancer

^a^Unpaired t-test for continuous variables and Chi-squared test for categorical variables were used for statistical comparison

**Fig. 1:**
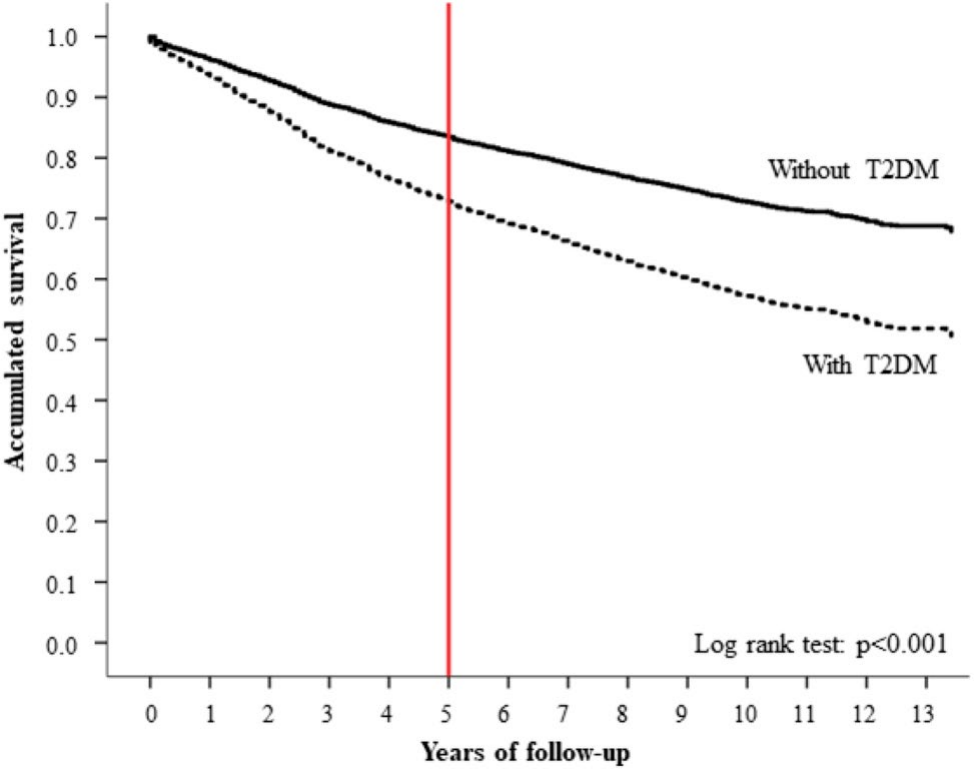
Unadjusted survival function of women with BC according to T2DM diagnosis

Overall, 1,299 (28.9%) women with BC died during complete follow-up and 785 (17.5%) did so during the first five years after BC diagnosis; therefore, the five-year survival rate was 82.5%. Compared to women without T2DM, the death rate was 59.3% higher in women with T2DM during the entire follow-up period (27.5% vs. 43.8%; *p* < 0.001) and 55.7% during the first 5 years (16.7% vs. 26.0%; *p* < 0.001). Crude survival curves according to T2DM diagnosis are shown in Fig. 1.

Women with coexisting BC and T2DM had a higher mortality risk when compared with those without T2DM: the adjusted hazard ratio (aHR) for overall mortality was 1.22 (95% CI 1.03–1.44) and 1.21 for five-year mortality (95% CI 0.98–1.51) (Table 2). When women were classified depending on whether the T2DM had been diagnosed before or after BC, a statistically significant mortality risk was only found among women with T2DM diagnosed before BC (aHR: 1.24; 95% CI 1.03–1.50 for overall mortality, and aHR: 1.26; 95% CI 1.00–1.60 for five-year mortality) (Table 3). Furthermore, in the analysis using three categories according to the time of T2DM diagnosis, the mortality risk was restricted to women with T2DM diagnosed > 5 years before BC (aHR: 1.31; 95%CI 1.01–1.69 for overall mortality, and aHR: 1.37; 95% CI 1.00–1.91 for five-year mortality) (Supplementary Table S1). In the ancillary analysis, we found that the excess of mortality risk associated with T2DM was only among women aged < 70 years old at BC diagnosis (Table 4).

**Table 2:** Risk of overall and five-year mortality according to T2DM diagnosis

	No T2DM	T2DM
Participants, n	4,105	388
Overall mortality		
Persons-years, *n*	35,789	3,049
Deaths, *n* (%)	1,129 (27.5)	170 (43.8)
Crude HR (95% CI)	1.00	1.76 (1.49–2.07)
Adjusted HR (95% CI)^a^	1.00	1.22 (1.03–1.44)
5-year mortality		
Persons-years, *n*	18,660	1,649
Deaths (%)	684 (16.7)	101 (26.0)
Crude HR (95% CI)	1.00	1.67 (1.35–2.06)
Adjusted HR (95% CI)^a^	1.00	1.21 (0.98–1.51)

*T2DM* type 2 diabetes mellitus, *HR* hazard ratio, *CI* confidence interval

^a^Cox proportional-hazards model adjusted for age at BC diagnosis (< 50, 50–69, ≥ 70), hospital size (< 500, ≥ 500 beds), BC suspected via screening (yes/no), multiple cancer (yes/no), BC stage (0-I, II, III-IV, not applicable, unknown), BC histology (non-invasive ductal carcinoma, invasive ductal carcinoma, non-invasive lobular carcinoma, invasive lobular carcinoma, other ductal and lobular, other types) BC location (breast quadrants, central part of the breast, nipple, axillary extensions, contiguous sites, unspecified sites) and types of treatments, including surgery (yes/no), chemotherapy (yes/no), radiotherapy (yes/no), hormone therapy (yes/no), immunotherapy (yes/no), targeted therapy (yes/no), and others (yes/no)

**Table 3:** Risk of overall and five-year all-cause mortality according to time of T2DM diagnosis

		Time of T2DM diagnosis	
	No T2DM	Before BC	After BC
Participants, *n*	4,105	159	96
Overall mortality			
Persons-years, *n*	35,789	2,189	860
Deaths (%)	1,129 (27.5)	133 (45.6)	37 (38.5)
Crude HR (95% CI)	1.00	1.89 (1.58–2.27)	1.41 (1.01–1.95)
Adjusted HR (95% CI)^a^	1.00	1.24 (1.03–1.50)	1.13 (0.81–1.57)
5-year mortality			
Persons-years, n	18,660	1,221	428
Deaths	684 (16.7)	82 (28.1)	19 (19.8)
Crude HR (95% CI)	1.00	1.82 (1.44–2.29)	1.24 (0.79–1.96)
Adjusted HR (95% CI)^a^	1.00	1.26 (1.00–1.60)	1.04 (0.66–1.65)

*T2DM* type 2 diabetes mellitus, *BC* breast cancer, *HR* hazard ratio, *CI* confidence interval

^a^Cox proportional-hazards model adjusted for age at BC diagnosis (< 50, 50–69, ≥ 70), hospital size (< 500, ≥ 500 beds), BC suspected via screening (yes/no), multiple cancer (yes/no), BC stage (0-I, II, III-IV, not applicable, unknown), BC histology (non-invasive ductal carcinoma, invasive ductal carcinoma, non-invasive lobular carcinoma, invasive lobular carcinoma, other ductal and lobular, other types) BC location (breast quadrants, central part of the breast, nipple, axillary extensions, contiguous sites, unspecified sites) and types of treatments, including surgery (yes/no), chemotherapy (yes/no), radiotherapy (yes/no), hormone therapy (yes/no), immunotherapy (yes/no), targeted therapy (yes/no) and others (yes/no)

**Table 4:** Risk of overall and five-year all-cause mortality according to T2DM diagnosis and stratified by age of BC diagnosis

	No T2DM	T2DM
Participants, n	4,105	388
Overall mortality		
< 50 years; adjusted HR (95% CI)^a^	1.00	2.38 (1.04–5.48)
50–69 years; adjusted HR (95% CI)^a^	1.00	1.65 (1.20–2.27)
≥ 70 years; adjusted HR (95% CI)^a^	1.00	1.04 (0.85–1.27)
5-year mortality		
< 50 years; adjusted HR (95% CI)^a^	1.00	2.40 (0.86–6.71)
50–69 years; adjusted HR (95% CI)^a^	1.00	1.57 (0.96–2.52)
≥ 70 years; adjusted HR (95% CI)^a^	1.00	1.10 (0.86–1.42)

*T2DM* type 2 diabetes mellitus, *BC* breast cancer, *HR* hazard ratio, *CI* confidence interval

^a^Cox proportional-hazards model adjusted for age at BC diagnosis (< 50, 50–69, ≥ 70), hospital size (< 500, ≥ 500 beds), BC suspected via screening (yes/no), multiple cancer (yes/no), BC stage (0-I, II, III-IV, not applicable, unknown), BC histology (non-invasive ductal carcinoma, invasive ductal carcinoma, non-invasive lobular carcinoma, invasive lobular carcinoma, other ductal and lobular, other types) BC location (breast quadrants, central part of the breast, nipple, axillary extensions, contiguous sites, unspecified sites) and types of treatments, including surgery (yes/no), chemotherapy (yes/no), radiotherapy (yes/no), hormone therapy (yes/no), immunotherapy (yes/no), targeted therapy (yes/no) and others (yes/no)

## Discussion

This survival analysis conducted among a cohort of 4,493 Spanish women with BC, showed that coexisting T2DM was associated with higher mortality after a mean of 8.66 years of follow-up. Moreover, a higher risk of mortality was seen among women with a longer history of T2DM (diagnosed > 5 years before BC) and in those with a younger age at BC diagnosis.

The five-year overall survival rate after BC in our cohort (82.5%) was consistent with findings from other studies involving Spanish populations. Chirlaque et al., using data from nine population-based registries, reported a five-year survival rate of 82.8% among women diagnosed between 2000 and 2007 [5]. Similarly, Baeyens-Fernández et al., found a five-year survival rate in Granada (Spain) of 83.7% in a cohort of women with a BC diagnosed in 2010–2012 (*n* = 8.502) [22]. Nevertheless, the overall survival rate after BC was lower than Northern European countries, the USA or Australia [23, 24], probably due to a socioeconomic gap in favor of these countries, which conditions more innovative treatment options, better established screening programs and more advanced diagnoses [25]. The lower survival rate in Spain is important to understand our findings related to the lower survival of Spanish women with BC and T2DM when compared with other studies. For instance, Maskarinec et al., in a survival analysis of a multiethnic cohort derived from cancer registries in Hawaii and California, found that 79.1% of women with T2DM were alive five years after BC diagnosis [17], representing a survival rate of approximately 5 percent higher than our cohort. Nevertheless, given that the study by Maskarinec et al., excluded women under 45 years of age at BC diagnosis [17], which is the age group with higher mortality rates after BC, according to our results, their survival rate may have been overestimated.

Although we found lower survival rates in our cohort after BC both for women with and without T2DM, the magnitude of the association between T2DM status and the risk of death could have been similar to other studies. However, the risk of death in women with BC associated to T2DM status was lower than reported in most studies, suggesting that T2DM could be a less important predictor of death for women with BC in Spain compared to other countries. In 2016, Zhao and Ren published a meta-analysis of 17 studies involving 48,315 women with BC from North America, Europe, and Asia [13]. The pooled adjusted HR of all-cause death was 1.51 (95% CI 1.34–1.70) and 1.46 (95% CI 1.21–1.76) when analyses were restricted to the eight studies with follow-up > 5 years, as in our study. More recently, Baglia et al., using data from participants of two population-based cohort studies in Shanghai, found an adjusted HR of 1.56 (95% CI 1.01–2.43) for all-cause death over a median follow-up of 3.4 years after BC diagnosis [26].

As in previous studies, we observed that the excess risk of mortality among women with BC and T2DM was dependent on the duration of T2DM. Thereby, the elevated all-cause mortality was only found for BC patients with longer T2DM duration. This finding may be attributed to the deleterious effect of common long-term T2DM complications. Luo et al., [27] and Maskarinec et al., [17], using a cut-off point of 7 years to define long T2DM duration, found a HR for overall mortality of 1.32 (95% CI 1.06, 1.66) and 1.27 (95% CI 1.07–1.49), respectively, which are almost equal to our figures. Moreover, Lega et al., [28] reported a HR of 1.28 (95% 1.15–1.43) using the same cut-off point for T2DM duration as in our study (five years), however, their analysis only involved women with stage III BC.

The higher mortality of women with BC and T2MD compared to women with only BC may be due to the long-term health effects of T2DM, that include deaths related to renal complications, and cardiovascular and cerebral vascular diseases [29, 30]. In addition, the interaction of BC and T2DM could be based on certain biological mechanisms that may help explain the effect of T2DM on survival of women with BC. Tumor development usually produces chronic inflammation, an increase of insulin growth factor and hyperinsulinemia [31, 32], which can lead to BC with worse prognosis [33]. Nevertheless, given that there were no differences in BC characteristics between women with or without T2DM from our cohort -even stage 0-I was more frequent among women with T2DM- the higher risk of death in women with T2DM was probably due to health conditions related to T2DM rather than to the effects of BC decreasing survival. Although we have no information about the cause of death, the majority of studies found that T2DM greatly increased the risk of death from other causes, and to a lower extent (or had no effect on) the risk of death for BC [17, 27, 28, 34, 35]. Moreover, this interpretation is coherent with the finding related to longer duration of T2DM, which may be an indicator not only of higher probability of vascular complications in damaged organs, but also of greater disease severity, higher accumulation of antidiabetic treatments and increased probability of uncontrolled T2DM. Consequently, it should be recognized that adjusting for types of T2DM treatment and glucose control could have changed our findings, as it would have enabled us to classify women according to disease severity and therapeutic management. Another possible explanation for the higher risk of mortality among women with T2DM lies in the differences in BC treatment in our data series. Compared to women without T2DM, those with T2DM were treated with significantly less intensity, which could lead to lower survival rates. It cannot be ruled out that women with preexisting T2DM, and thus with compromised cardiometabolic health, may have received lower BC treatment intensity due to their worse baseline health status. In any case, the differences in therapeutic approaches of BC according to T2DM status and their potential effect on survival merits further research.

Finally, the inverse dose–response effect of the age at BC diagnosis on the association between T2DM and the risk of death could be conditioned by several factors. First, young women with T2DM and BC usually have tumors with worse prognosis [14, 36]. Second, they could also have more treatment side effects and complications, especially related to BC surgery [37]. Third, there is a growing body of evidence suggesting that young-onset T2DM has worse glycemic and lipid control, and more complications compared to older onset T2DM patients [38]. In any case, this finding underscores the need for a rigorous follow-up of BC complications and a more exhaustive metabolic control of T2DM among young women.

Our study has some important limitations that need to be considered when interpreting the findings. First, we lack information on anthropometric measures, which are well stablished predictors of adverse health outcomes and death both among individuals with BC and T2DM. Particularly, we have no measure of body fat, despite the fact that endocrine alternations caused by excessive adipose tissue can induce tumor progression, leading to a decrease in life expectancy. Moreover, weight gain is also common after BC treatments, with a consequent potential impact on T2DM control. In short, consideration should be given to the close relation of obesity with the T2DM/BC binomial, as adjusting for body weight would probably reduce or eliminate statistically significant differences in death rates between BC women with or without T2DM. Second, we also lack data on socioeconomic characteristics, lifestyle variables and comorbidity, although it is known that certain unhealthy behaviors, low education level and comorbid conditions commonly associated to T2DM may lead to poor overall health status. Thereby, we cannot disentangle the effects of T2DM related factors from the disease itself. Third, although we used a public administrative database that is very accurate to ascertain date of death, this database contains no information about the specific cause of death. Thus, our study was unable to determine whether T2DM increased the risk of death for BC or for other causes. Nevertheless, misclassification when assigning cause of death may be highly relevant when studying women with BC and T2DM. Patients who die shortly after BC diagnosis may be more likely to be assigned BC as cause of death, whereas women dying after BC remission may be more prone to be classified as having a cardiovascular cause of death, because of the well-known association of T2DM with cardiovascular mortality.

In conclusion, Spanish women with T2DM prior to BC had worse all-cause survival, particularly when BC was diagnosed before the age of 50. Our findings add consistency to the previous studies among BC women from several countries and ethnic groups. Nevertheless, considering the lack of information regarding certain relevant variables, including body weight, morbidity and T2DM treatment, caution is suggested when interpreting the study findings. Longitudinal research is needed to study whether the clinical and psychological impact of BC diagnosis disrupts T2DM management, affecting health status and decreasing survival rates.

## Supplementary Information

Below is the link to the electronic supplementary material.

Supplementary file1 (DOCX 18 kb)

## Data Availability

Public data from the state population-based cancer registry of Asturias (Spain) are available on reasonable request.
