# Supplementary material for: Type 2 Diabetes and all-cause mortality among Spanish women with breast cancer
Source: Cancer Causes Control. 2021 Dec 1;33(2):271–8. doi: 10.1007/s10552-021-01526-x (PMC8776668; doi:10.1007/s10552-021-01526-x)
Supplement: Supplementary file 1 — Supplementary file1 (DOCX 18 kb) [file 10552_2021_1526_MOESM1_ESM.docx]

**Supplementary Table 1**. Risk of overall and five-year all-cause mortality according to time of T2DM diagnosis

|  |  |  | **Time of T2DM diagnosis** | | |
| --- | --- | --- | --- | --- | --- |
|  |  | **No T2DM** | **>5 years before BC** | **0-5 years before BC** | **After BC** |
| Participants, n | | 4,105 | 133 | 159 | 96 |
| Overall mortality | |  |  |  |  |
|  | Persons-years, n | 35,789 | 955 | 1,234 | 860 |
|  | Deaths (%) | 1,129 (27.5) | 62 (46.6) | 71 (44.7) | 37 (38.5) |
|  | Crude HR (95% CI) | 1.00 | 2.04 (1.58-2.64) | 1.77 (1.39-2.26) | 1.41 (1.01-1.95) |
|  | Adjusted HR (95% CI)^a^ | 1.00 | 1.31 (1.01-1.69) | 1.19 (0.93-1.52) | 1.13 (0.81-1.57) |
| 5-year mortality | |  |  |  |  |
|  | Persons-years, n | 18,660 | 555 | 665 | 428 |
|  | Deaths | 684 (16.7) | 39 (29.3) | 43 (27.0) | 19 (19.8) |
|  | Crude HR (95% CI) | 1.00 | 1.97 (1.43-2.72) | 1.69 (1.23-2.32) | 1.24 (0.79-1.96) |
|  | Adjusted HR (95% CI)^a^ | 1.00 | 1.37 (1.00-1.91) | 1.17 (0.85-1.62) | 1.04 (0.66-1.65) |

T2DM: type 2 diabetes mellitus; BC: breast cancer; HR: hazard ratio; CI: confidence interval

^a^Cox proportional-hazards model adjusted for age at BC diagnosis (<50, 50-69, ≥70), hospital size (<500, ≥500 beds), BC suspected via screening (yes/no), multiple cancer (yes/no), BC stage (0-I, II, III-IV, not applicable, unknown), BC histology (non-invasive ductal carcinoma, invasive ductal carcinoma, non-invasive lobular carcinoma, invasive lobular carcinoma, other ductal and lobular, other types) BC location (breast quadrants, central part of the breast, nipple, axillary extensions, contiguous sites, unspecified sites) and types of treatments, including surgery (yes/no), chemotherapy (yes/no), radiotherapy (yes/no), hormone therapy (yes/no), immunotherapy (yes/no), targeted therapy (yes/no) and others (yes/no).
